# Writing for different readers

**DOI:** 10.7554/eLife.25408

**Published:** 2017-03-15

**Authors:** Peter Rodgers

**Keywords:** plain-language summaries, scientific publishing, public engagement

## Abstract

More could be done to make research papers readily understandable by the public.

One of the most compelling arguments for open access is that most research is funded by the public, so the fruits of that research should somehow be available to the public. If public money pays for research into the workings of cells, the causes of diseases or the possibility of life on other planets, then the public should be able to read the research papers that emerge from these projects. And increasingly they can, courtesy of the growth of open-access journals and, more recently, the rise of preprints. There has also been progress in other aspects of open science, such as open data and open-source software (McKiernan et al., 2016). However, there is still a long way to go before paywalls disappear and all research papers are made freely available at the time of publication.

But if we want to take the open agenda to its logical conclusion we must do more. It is not enough to give everyone access to every research paper and leave it at that: we have to make research open in other ways. We have to make an effort to communicate with readers outside the research community; we have to speak to pupils and teachers, to healthcare professionals and patients (and their families), to anyone and everyone who is interested in science and research. And we have to speak to them in their language, in the language of the news media and Wikipedia. We have to speak to them in plain language, not in the formal and formulaic prose found in most research papers; and we have to use verbs, not nouns, and to avoid words like characterization and facilitation that – while much loved and used by scientists – can stop a sentence or article dead in its tracks.

Activity in science communication – also known as the public understanding of science, public engagement, or science and society – has grown significantly over the past few decades: researchers in the field have their own journals, conferences and, of course, specialized vocabulary; universities, funding bodies and medical charities employ large numbers of press officers and outreach staff (although newspapers and magazines employ fewer science reporters); podcasts, blogs and social media have thrived; and there is a growing number of exhibitions, festivals and events in venues like pubs and cafes. Here, and in three related articles, we focus on a niche area within this rich tapestry – the plain-language summary of a research proposal or paper.

As explained in "An inside guide to eLife digests", we have been publishing plain-language summaries of eLife papers, called digests, since the journal was launched in 2012 (King et al., 2017). These summaries are typically between about 250 and 400 words long and appear immediately below the abstract (and at the top of the second page in the PDF version). The aim of the digest is threefold – to describe the background to the paper, to summarize the main findings, and to briefly discuss what might happen next – in language that an interested or motivated reader can understand. While eLife digests are primarily intended for readers outside the research community, a recent survey suggested that they are widely read by other researchers. And plain-language summaries can also be useful to authors when, for example, they need to explain their work in non-technical terms when applying for a fellowship or faculty position.

At first we prepared digests for all eLife papers. It did not matter if the paper was about ribosome-associated degradation or breast cancer, it contained a digest. It was difficult to write digests for some papers, especially if the action never left the cell, but we found a way. However, the continuous growth in the number of papers accepted for publication meant that in 2016 we had to, reluctantly, start publishing papers without digests.

Of course, eLife is not the only research journal to publish plain-language summaries of research papers. As reported in "Something for everyone", journals covering fields as diverse as autism, ecology and rheumatic diseases publish such summaries, as do the American Astronomical Society and the British Psychological Society (Shailes, 2017). One challenge facing all these bodies is to make their summaries readily discoverable by the intended readership.

The area of science with perhaps the greatest need for clear and accurate information about current research is medical research and, as described in "The value of a healthy relationship", medical charities and patient groups are very active in this arena (Kuehn, 2017). Some charities require researchers to include plain-language summaries with applications for funding, and others include patient representatives in the panels that evaluate funding applications.

There is nothing new in expecting academic researchers to communicate with the public.

There is actually nothing new in expecting academic researchers to communicate with the public: the founding document of the American Association of University Professors, published in 1915, states that one of the roles of an academic is to "impart the results of their own and their fellow-specialist's investigations and reflection, both to students and to the general public" (Sugimoto, 2016). And the challenge of communicating complex subject matter to a general audience is not unique to science. Last year, for example, Jonathan Fullwood of the Bank of England compared the readability of written outputs from five different sources: he found that reports and speeches from his employer and other banks were the least readable, and that political speeches were the most readable. The reason, he wrote, is that "those writing in the financial industry tend to use long words. They put those words in long sentences. And those sentences in long paragraphs" (Fullwood, 2016). It is hard to disagree, even if this article does contain one sentence that runs to 87 words.

Roughly two million research papers are published every year and, given our experiences on eLife, it would be extremely difficult to ensure that each one had a plain-language summary. However, there is definitely scope for more journals to offer the option of such summaries. Such a move would benefit authors, journals, other scientists and the world at large.
